# First person – Jacqueline Weidner

**DOI:** 10.1242/bio.051011

**Published:** 2020-02-17

## Abstract

First Person is a series of interviews with the first authors of a selection of papers published in Biology Open (BiO), helping early-career researchers promote themselves alongside their papers. Jacqueline Weidner is first author on ‘Hormones as adaptive control systems in juvenile fish’, published in BiO. Jacqueline conducted the research described in this article while a PhD student at the University of Bergen, Norway. She is now an assistant professor at the Western Norway University of Applied Sciences, Norway, investigating sexual selection and modelling of evolutionary patterns.


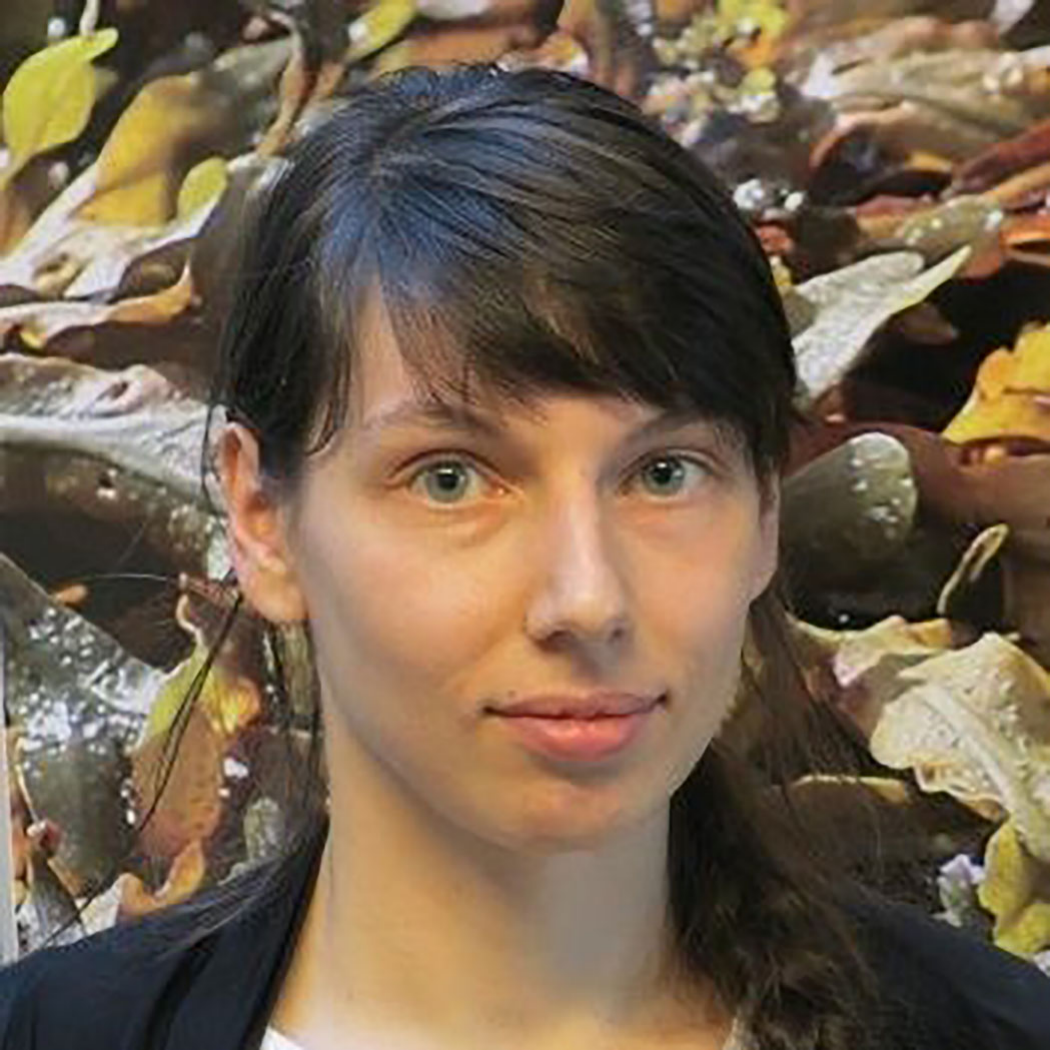


**Jacqueline Weidner**

**What is your scientific background and the general focus of your lab?**

During my first year as a biology student, I attended a course on evolution. Since then my interest in evolution has grown. In my master's thesis, I worked with sexual selection in Arctic char. For my PhD, I kept fish as model organisms and kept my interest in evolutionary patterns, but replaced empirical with theoretical work. The transition was hard, but a very valuable experience because of the great differences between the fields, their way of planning and designing experiments and writing articles. Today, I know we need both fields as complementary ways of thinking and doing science.

The research group I have been part of is diverse in terms of studied species and traits, ranging from selection in birds to vision in marine fish. What keeps them as a group is their interest in using theoretical models to describe ecological and evolutionary patterns.

**How would you explain the main findings of your paper to non-scientific family and friends?**

Growth is an essential aspect of life. All individuals have to grow to reach a size at which they can reproduce and get offspring. However, there are differences in growth speed between individuals of one species. These can be due to measurable factors such as access to food or temperature, i.e., energy requirements for growth cannot be met when food gets scarce. In addition to these more-or-less obvious growth restrictions, there are considerations and decisions individuals have to make during their growth phase. Such considerations can focus on whether to eat or hide for predators. When weighting the different options, individuals have to assess the amount of food they can find, the risk of being caught by a predator and so on. In my current work, we use a model to investigate such challenges in a growing fish. The fish is a simplified model using hormones to translate environmental conditions to signals comprehensible by the body. Those signals also regulate energy distribution in the body, e.g. energy used for growth or metabolism. Results from the model indicate how growth strategies could change under certain environmental scenarios and how these changes are mediated by hormones.

**What are the potential implications of these results for your field of research?**

A main message from the paper is the need for more interdisciplinary work. In our article, we encourage physiologists, modelers and evolutionary biologists to work together. While models can help to sort and focus thoughts and ideas, and to simplify, we never should forget to relate model results to the real world and organisms in nature. We sorted out some interesting patterns. Now, they need to be integrated into empirical research for both validation and improvement of the model. I think a collaboration with physiologists is an important next step.

“A main message from the paper is the need for more interdisciplinary work.”

**What has surprised you the most while conducting your research?**

I have been surprised by the complexity of systems in our bodies; the huge number of hormones, neurotransmitters and other molecules acting simultaneously to make bodies function in constantly changing environments. When diving into literature, which was necessary during model development, I read about an enormous number of signal carriers and their many different pathways for signal transduction through receptors, binding molecules and so on. Having said that, it is even more surprising how difficult it is to reduce this complexity when building a model. All the detailed knowledge about endocrinal systems has to be sorted, summarized and simplified. One of the big questions in this phase was how to prioritize and integrate signaling processes and reduce them to fit the model. A model is never and never could be a copy of nature because of this complexity, but can be a tool for assisting our thought processes and so the simplification of natural processes into model pathways is important.

**Figure:**
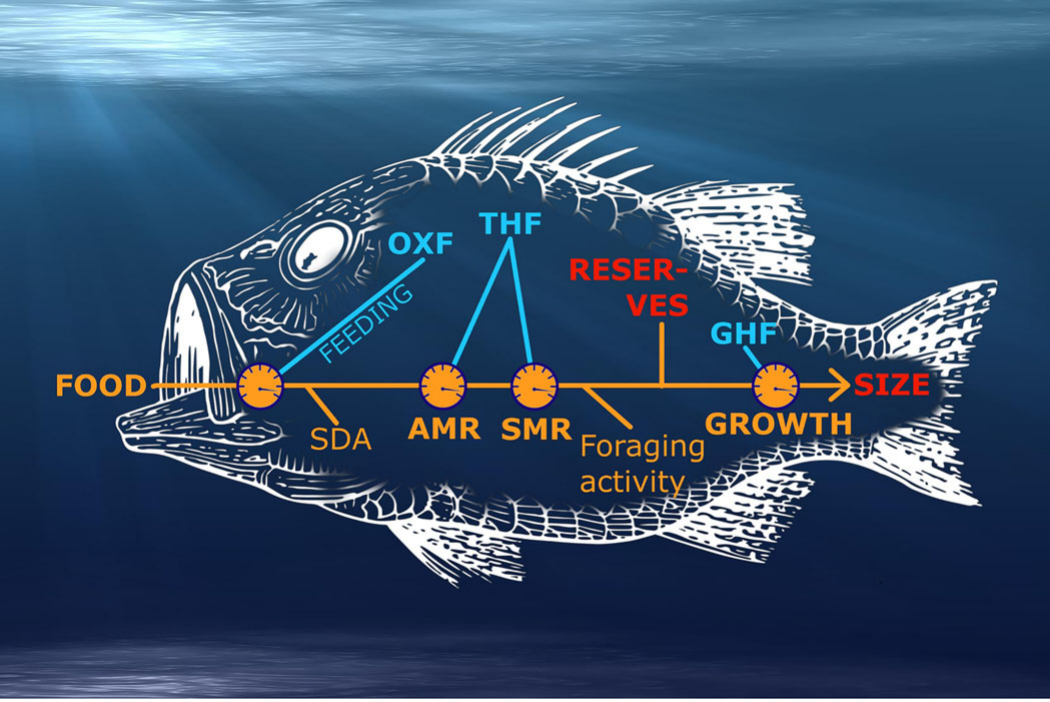
**The proximate and the ultimate – how hormones and survival affect adaptations of growth rates in juvenile fish.**

“I have been surprised by the complexity of systems in our bodies.”

**What changes do you think could improve the professional lives of early-career scientists?**

Early-career scientists often face an insecure future with short-term contracts. This concerns both their career and personal life and maybe makes young great minds leave academia. It also makes life harder for those who want to combine family and work, which is extremely sad. Maybe the reason for this is an obsolete tradition for valuing established scientists much higher than early-career scientists, e.g. by counting the number of publications, invited speeches and approved funding proposals. In media, children often are referred to as ‘our future’. Could we adopt this into science? Making early-career scientists ‘our future scientists’? This would change the academic system of values without devaluating the knowledge and experience of today's established scientists, but improving early-career scientist status and, in the long run, their working situation.

**What's next for you?**

I do not know. Right now, I have a temporary position as an assistant professor, but my PhD is not finished. Allocating time between my current position, PhD work and my family moves focus away from dreaming or planning future positions. I love to teach and I really would like to combine teaching and research in a future job. I also miss working with research questions related to sexual selection, which is a fascinating topic. The chance of getting a permanent position seems to be tiny and moving between temporary positions at different places is not an option when having children. I am flexible and eager to try out new things, which makes finding a job easier but leaves the job search an open-ended process.
